# Editorial: Myocarditis and inflammatory cardiomyopathies: diagnosis, treatment and future directions

**DOI:** 10.3389/fcvm.2023.1321494

**Published:** 2023-11-14

**Authors:** Piero Gentile, Entela Bollano, Giuseppe Vergaro, Emanuele Bobbio

**Affiliations:** ^1^ De Gasperis Cardio Center, Niguarda Hospital, Milan, Italy; ^2^ Department of Cardiology, Sahlgrenska University Hospital, Gothenburg, Sweden; ^3^ Institute of Medicine, Sahlgrenska Academy at the University of Gothenburg, Gothenburg, Sweden; ^4^ Department of Cardiology, Fondazione Toscana Gabriele Monasterio, Pisa, Italy

**Keywords:** myocarditis acute and fulminant, heart failure, sarcoidosis, systematic review, imaging, cardiomyopathy

**Editorial on the Research Topic** Myocarditis and inflammatory cardiomyopathies: diagnosis, treatment and future directions

Myocarditis is defined as an inflammatory condition affecting the myocardium and is typically diagnosed based on the Dallas criteria (1). These criteria encompass histological factors, such as the presence of an inflammatory infiltrate combined with degeneration and necrosis, as well as immunological and immunohistochemical parameters (1). Determining the exact incidence of acute myocarditis (AM) is challenging due to the reliance on endomyocardial biopsies for precise diagnosis. As a rough estimate, AM was believed to impact approximately 22 out of every 100,000 individuals before the beginning of the COVID-19 pandemic in 2020 (2).

The clinical presentation of this condition encompasses a variety of signs and symptoms, such as chest pain, heart failure, ventricular arrhythmias, or shortness of breath (3, 4). Although most patients presents with mild symptoms, myocarditis can also lead to acute heart failure and life-threatening arrhythmias or cardiogenic shock (3, 4).

Based on contemporary guidelines, cardiac magnetic resonance (CMR) plays a pivotal role in the diagnostic work-up of suspected AM, however, endomyocardial biopsy (EMB) is still considered the gold standard for the diagnosis (5, 6). Despite its invasive character and the relatively low sensitivity, the information derived from EMB is fundamental for identifying the mechanisms and deciding therapy and should be used for select patients (4).

To gain insight into myocarditis and inflammatory cardiomyopathies, the present Research Topic, entitled “Myocarditis and Inflammatory Cardiomyopathies: Diagnosis, treatment and future directions” aggregated relevant and original research studies, reviews, clinical cases and meta-analysis that explore this pathophysiological entity.

We identified two original research papers that studied clinical and CMR predictors in patients with myocarditis. Firstly, Cannatà et al. conducted an analysis involving 199 patients with CMR-confirmed AM and found that AM cases presenting with life-threatening arrhythmias were associated with a higher risk of adverse events. In their registry three-quarters of patients with AM presented with chest pain, which was associated with a benign prognosis. A protective value of a chest pain as presenting manifestation was also observed by Bohbot et al. The Authors studied clinical and CMR predictors in 388 hemodynamically stable patients with AM. They found that the absence of oedema, reduced ejection fraction, and the extent of late gadolinium enhancement were all associated with early adverse outcomes.

Patients with myocarditis following the administration of mRNA SARS-CoV-2—vaccination were studied by Shiyovich et al. and Schroth et al. Shiyovich et al. specifically focused on adolescents aged 12–15 years who experienced myocarditis following the administration of the BNT162b2 mRNA COVID-19 vaccine. Their study revealed that the CMR imaging findings, consistent with the clinical course, resembled those observed in older patients. These findings suggested relatively mild myocarditis in this population, potentially indicating a favourable clinical course and outcomes. On the other hand, Schroth et al. conducted a study involving 59 patients (80% males, mean age 29 years) with CMR-diagnosed mild myocarditis resulting from mRNA SARS-CoV-2 vaccinations. This study aimed to identify predictors of persistent symptoms in individuals with vaccine-related myocarditis. A significant portion of their patients (24%) reported enduring symptoms, including chest pain (67%), dyspnoea (58%), and an increasing occurrence of fatigue (42%) and palpitations (17%). Schroth et al. observed that patients with persistent symptoms were predominantly females and older individuals.

In their review, Garg et al. investigated CMR findings in cases of myocarditis associated with COVID and SARS-CoV-2 vaccination. They observed similarities in myocardial injury patterns among acute disease, post-COVID, and SARSCoV-2 vaccination, suggesting a non-specific underlying pathophysiology. The authors posit that most instances of myocardial inflammation may arise from generic inflammatory injury rather than direct viral damage.

To gain a more comprehensive understanding of myocarditis pathogenesis and provide valuable guidance for drug development and clinical treatment, Xuan et al. conducted a study exploring the intricate interactions among cardiomyocytes (CMs), cardiac fibroblasts (CFs), and endothelial cells (ECs). CFs and ECs are susceptible to pathogen infection and can release immunologically active substances, contributing to the inflammatory response. Additionally, CFs influence the fibrosis process and the long-term prognosis of the heart through changes in the extracellular matrix (ECM). Furthermore, their interactions with CMs may either enhance or hinder the disease progression.

In this research topic focusing on myocarditis and inflammatory cardiomyopathies, we encounter three manuscripts addressing rare forms of these conditions. Firstly, there is a review on cardiac sarcoidosis, and secondly, two clinical cases discuss myocarditis associated with immune checkpoint inhibitors (ICIs) and tuberculous myocarditis.

Sarcoidosis is an inflammatory disease characterized by multisystem non-caseating granulomas, which most commonly affects the lungs (7, 8). The development of sarcoidosis-associated PH (SAPH) significantly increases mortality in individuals with sarcoidosis. Zhang et al. conducted a meta-analysis of 25 studies spanning 12 countries to determine the prevalence of SAPH in general and advanced sarcoidosis populations. Their findings, based on right heart catheterization, revealed a pooled prevalence of SAPH at 6.4% (95% CI: 3.6%–9.1%) in the general sarcoidosis population, with pre-capillary PH at 6.5% (95% CI: 2.9%–10.2%).

A rare form of myocarditis is associated to the use of ICIs. A case of pembrolizumab-induced myocarditis following COVID-19 infection was described by Nishiyama et al. It’s worth noting that in patients with acute myocarditis, elevated myocardial markers and electrocardiographic changes may precede clinical symptoms, underscoring the importance of regular myocardial marker measurements and electrocardiographic monitoring during ICI administration (9).

Zhang et al. described a rare case of tuberculous myocarditis (TM), an exceedingly rare manifestation of Mycobacterium tuberculosis (TB) infection. In this patient, a definitive diagnosis of TM was established, and histopathological findings were consistent with sinus node involvement, as determined through autopsy results. Furthermore, the authors provide an insightful overview of the challenges associated with diagnosing myocardial TB.

The objective of this Research Topic was to address significant knowledge gaps in this protean clinical entity. Substantial efforts need to be made to identify the precise underlying causes for individual patients in various situations, enabling the customization of targeted therapies.
